# DET1 and COP1 Modulate the Coordination of Growth and Immunity in Response to Key Seasonal Signals in *Arabidopsis*

**DOI:** 10.1016/j.celrep.2018.08.096

**Published:** 2018-10-02

**Authors:** Sreeramaiah N. Gangappa, S. Vinod Kumar

**Affiliations:** 1 Cell and Developmental Biology Department, John Innes Centre, Norwich NR4 7UH, UK

**Keywords:** *Arabidopsis*, immunity, photoperiod, temperature, DET1, COP1, PIF4, environmental signal integration

## Abstract

Plant growth and development and outcomes of plant-microbe interactions are defined by coordinated responses to seasonal signals. The mechanisms that control the coordinated regulation of growth and immunity are not well understood. Here, we show that a common signaling module integrates environmental signals, such as photoperiod and temperature, to regulate the growth-defense balance. Key light-signaling components De-Etiolated 1 (DET1) and Constitutive Photomorphogenic 1 (COP1) negatively regulate immunity and are essential for immune modulation by photoperiod and temperature. Our results show that this is regulated by the transcription factor Phytochrome Interacting Factor 4 (PIF4), suggesting that the DET1/COP1-PIF4 module acts as a central hub for the control of growth and immunity in response to seasonal signals. These findings provide a regulatory framework for environmental signal integration.

## Highlights

• Photoperiod modulates immunity in *Arabidopsis*
• DET1 and COP1 mediate the coordination of growth and immunity
• PIF4 plays a key role in the modulation of immunity by photoperiod
• The DET1/COP1-PIF4 module controls growth and immunity in response to seasonal signals

## Graphical Abstract

**Figure:**
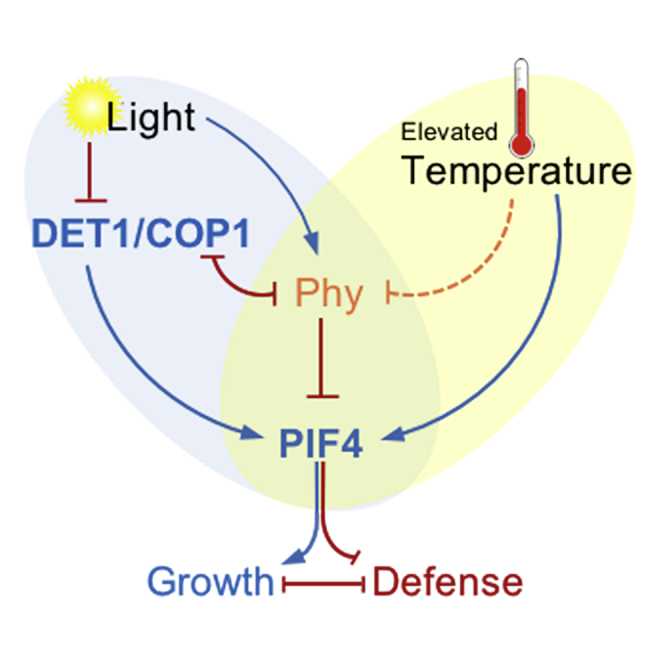

## Teaser

In plants, adaptive traits such as growth and immunity are strongly influenced by the environment. How multiple seasonal signals are integrated is not well understood. Gangappa and Kumar show that a common signaling module comprising DET1, COP1, and PIF4 coordinates growth and immunity in response to key seasonal signals.

## Introduction

Perception and integration of seasonal signals and diurnal fluctuations into biological processes define plant phenology and adaptation (Møller and Chua, 1999, Vert and Chory, 2011). Notably, key seasonal signals such as photoperiod and temperature have been shown to strongly influence plant processes such as growth and development, as well as plant-pathogen interactions (Alcázar and Parker, 2011, Ballaré, 2014, Dietrich et al., 1994, Hua, 2013). Specifically, photoperiod strongly influences plant phenological and physiological responses (Fraser et al., 2016, Song et al., 2013) and it has been well understood (Song et al., 2013). Moreover, it plays a key role in modulating plant defense responses that are exemplified by the modulation of lesion-mimic mutant phenotypes (Dietrich et al., 1994, Chaouch et al., 2010) and resistance to pathogens (Roden and Ingle, 2009, Hua, 2013). In spite of the effect of photoperiod on conditioning immunity, the signaling hierarchy and molecular mechanisms that dictate this remain unknown. Beyond fundamental biology, elucidating the underlying mechanisms that modulate plant immunity in response to diurnal and seasonal signals is of great significance for sustainable productivity, especially in the wake of climate change (Battisti and Naylor, 2009, Gornall et al., 2010).

Phytochrome Interacting Factor 4 (PIF4), a basic helix-loop-helix (bHLH) transcription factor, plays a key role in promoting growth in response to warm ambient temperatures (Koini et al., 2009, Kumar et al., 2012) and in coordinating growth and defense (Gangappa et al., 2017, Paik et al., 2017). In recent years, PIFs have emerged as a hub for environmental signaling and response (Leivar and Monte, 2014, Leivar and Quail, 2011). PIF-mediated growth is defined by an external coincidence mechanism, which through coordinated control of PIF function defines diurnal growth (Niwa et al., 2009, Nomoto et al., 2012, Nozue et al., 2007). We have recently shown that PIF4-mediated thermosensory elongation growth is photoperiod dependent (Gangappa and Kumar, 2017) and requires the action of De-Etiolated 1 (DET1) and Constitutive Photomorphogenic 1 (COP1). DET1 and COP1 are two key negative regulators of light signaling that act to promote PIF4 function through maintaining PIF4 protein levels (Gangappa and Kumar, 2017). In light of this, we hypothesized that seasonal signals could be integrated through a shared signaling module for the coordinated control of growth and defense responses. Here, we show that light and temperature signals modulate growth and immunity through a common signaling circuitry consisting of DET1 and COP1 and their downstream regulators such as PIF4. We show that defense gene expression and disease resistance in *Arabidopsis* is strongly influenced by day length, in which PIF4 plays a key role. Consistent with the role of DET1 and COP1 in promoting PIF4 function, we found that PIF4 also plays an important role in suppressing immunity in shorter photoperiods. Moreover, DET1 and COP1 were found to be essential for elevated temperature-mediated suppression of defense responses. Collectively, our study highlights the integration of light and temperature signals through the DET1/COP1-PIF4 module to coordinate growth and immunity.

## Results and Discussion

### Disease Resistance Is Influenced by Photoperiod

**Figure 1:**
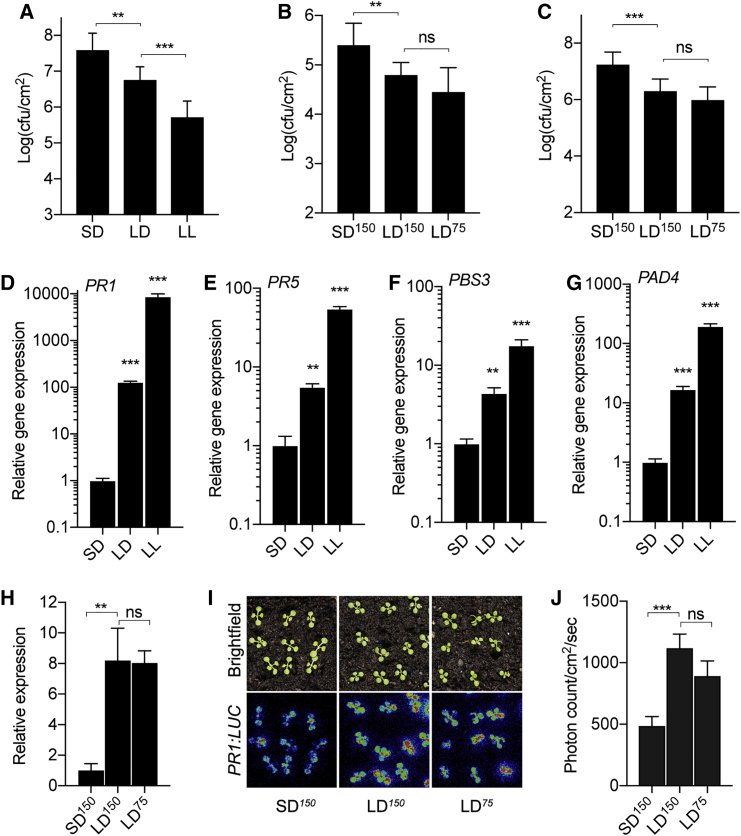
Plant Immunity Is Modulated by Photoperiod (A) Resistance to *Pto* DC3000 of 3-week-old SD-, LD-, and constant light (LL)-grown Col-0 (n ≥ 4) as a function of day length. (B and C) Resistance phenotype of 3-week-old (B) and 12-day-old (C) Col-0 plants grown under SD^150^ (150 μmol m^−2^ s^−1^), LD^150^ (150 μmol m^−2^ s^−1^), and LD^75 (^75 μmol m^−2^ s^−1^) conditions to *Pto* DC3000. (D–G) Gene expression of *PR1* (D), *PR5* (E), *PBS3* (F), and *PAD4* (G) in 12-day-old Col-0 seedlings grown on soil under SD, LD, and LL conditions (n = 3). (H) Expression of *PR1* in 12-day-old Col-0 seedlings grown under SD^150^ (150 μmol m^−2^ s^−1^), LD^150^ (150 μmol m^−2^ s^−1^), and LD^75^ (75 μmol m^−2^ s^−1^) conditions in Murashige and Skoog (MS) plates (n = 3). (I) Expression of *PR1:LUC* from plants grown under SD^150^ (150 μmol m^−2^ s^−1^), LD^150^ (150 μmol m^−2^ s^−1^), and LD^75^ (75 μmol m^−2^ s^−1^) conditions. Seedlings were grown in SD^150^ 3 days before being shifted to LD (LD^150^ and LD^75^) for an additional 3 days and imaged for luminescence. (J) Normalized luminescence of *PR1:LUC* in seedlings (n ≥ 40) shown in (H). The data shown are representative of three independent experiments. Values shown are means ± SDs. Asterisks indicate statistically significant difference by Student’s t test (^∗∗^p ≤ 0.01, ^∗∗∗^p ≤ 0.001) from Col-0 or between indicated pairs of conditions or genotypes. cfu, colony forming unit; ns, not significantly different.

To dissect the influence of light on defense responses, we studied the disease resistance of *Arabidopsis* to the bacterial pathogen *Pseudomonas syringae* pv. *tomato* (*Pto*) DC3000 at different photoperiods. Three-week-old *Arabidopsis* wild-type (Col-0) plants grown at short-day (SD; 8 hr light/16 hr dark), long-day (LD; 16 hr light/8 hr dark), and constant light (LL; 24 hr light) were challenged with *Pto* DC3000 by spray inoculation (A_600_ = 0.002). Three days post inoculation, plants grown at LD showed increased resistance, as observed by reduced bacterial growth as opposed to those grown at SD (Figure 1A). In addition, plants grown at LL showed significantly enhanced resistance as opposed to those grown at LD (Figures 1A and S1A), confirming that plant immunity and disease resistance to *Pto* DC3000 is strongly influenced by day length. Notably, susceptibility to the bacterial pathogen increased with shortening of day length.

Because the above experiments were conducted with the same light intensity levels (photosynthetic photon flux density of 150 μmol m^−2^ s^−1^), plants grown at 16 hr LD would receive twice as much photosynthetically active radiation (PAR) as those grown at 8 hr SD. To check whether the increased resistance at 16 hr LD was due to PAR or day length per se, we compared the disease resistance of plants grown at LD with 75 m^−2^ s^−1^ (LD^75^) and 150 m^−2^ s^−1^ (LD^150^) along with SD at 150 μmol m^−2^ s^−1^ (SD^150^) light intensity. While both LD^75^- and LD^150^-grown plants showed enhanced resistance compared to SD^150^, no significant differences were observed between LD^75^ and LD^150^ (Figure 1B), suggesting that altered photon flux is not the primary cause of increased resistance at LD. In addition, to rule out the possibility that the observed effect of day length could be a likely consequence of photoperiod-induced developmental changes, we analyzed 12-day-old seedlings for resistance to *Pto* DC3000 and found strong photoperiod-dependent modulation, as observed in adult plants (Figure 1C), highlighting the strong influence of photoperiod on plant immunity. Further supporting this, we found that the expression of key defense genes such as *PATHOGENESIS RELATED 1* (*PR1*), *PR5*, *AVRPPHB SUSCEPTIBLE 3* (*PBS3)*, and *PHYTOALEXIN DEFICIENT 4* (*PAD4*) are significantly elevated with increased photoperiod (Figures 1D–1G). The expression of *PR1:LUC* was also found to be higher with increased photoperiod (Figures S1B and S1C). As shown above, increased *PR1* expression at LD was not dependent on altered PAR (Figures 1H–1J). Collectively, these results show that similar to disease resistance, photoperiod-dependent modulation of defense gene expression by light in our experimental system is due to the length of the illuminated period rather than the likely metabolic consequences associated with photoassimilation.

### DET1/COP1 Signaling Mediates Photoperiod Modulation of Immunity

**Figure 2:**
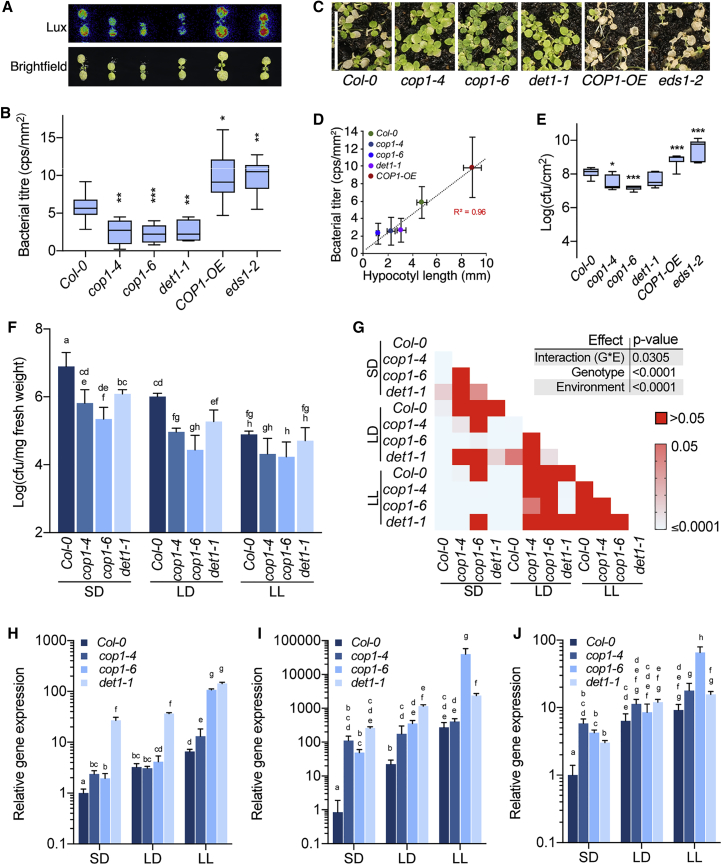
DET1 and COP1 Negatively Regulate Immunity in Response to Photoperiod (A) Luminescence image showing the disease-resistance phenotype of 10-day-old Col-0, *cop1-4*, *cop1-6*, *det1-1*, and *COP1-OE* to *Pto* DC3000-lux (A_600_ = 0.002) in SD *eds1-2* is used as a susceptible control. (B) Normalized luminescence data from seedlings shown in (A) (n = 8). (C) Representative image of Col-0, *cop1-4*, *cop1-6*, *det1-1*, *COP-OE*, and *eds1-2* 10 days post-spray inoculation with *Pto* DC3000-lux. (D) Correlation of hypocotyl elongation and disease susceptibility in indicated genotypes. (E) Resistance to *Pto* DC3000 (A_600_ = 0.002) in 4-week-old Col-0, *cop1-4*, *cop1-6*, and *det1-1* and *COP1-OE* grown in 22°C SD (n = 8). (F) Resistance to *Pto* DC3000 (A_600_ = 0.002) of 12-day-old Col-0, *cop1-4*, *cop1-6*, and *det1-1* plants grown in 22°C under SD, LD, and LL conditions (n = 8). (G) Two-way ANOVA followed by Tukey’s honest significant difference (HSD) test of data from (F). (H–J) Expression of *PR1* (H), *PBS3* (I), and *PAD4* (J) in Col-0, *cop1-4*, *cop1-6*, and *det1-1* mutants from 1-week-old SD-, LD-, and LL-grown seedlings (n = 3). Data shown are the means ± SDs of three biological replicates. The data shown are representative of three independent experiments; data points with the same letters are statistically not significant based on two-way ANOVA followed by Tukey’s multiple comparison test (p < 0.05). Asterisks indicate statistically significant (Student’s t test; ^∗^p ≤ 0.05, ^∗∗^p ≤ 0.01, ^∗∗∗^p ≤ 0.001) difference from Col-0.

We have recently shown that the key light-signaling components COP1 and DET1 play an important role in photoperiod-dependent elongation growth (Gangappa and Kumar, 2017). Given the apparent antagonism between growth and immunity, we hypothesized that the DET1/COP1 signaling module could also have a novel role in the photoperiod-dependent modulation of immunity. We screened the corresponding mutants for resistance using a luminescent *luxCDABE*-tagged *P. syringae* strain, *Pto*-DC3000-lux (Fan et al., 2008), in 10-day-old SD-grown seedlings. Three days after spray inoculation, *cop1-4*, *cop1-6*, and *det1-1* mutants showed significantly reduced bacterial growth, as shown by luminescence, compared to that of Col-0 (Figures 2A and 2B). Complementary to this, a transgenic line overexpressing *COP1* (*COP1-OE*) (Holm et al., 2001) showed increased susceptibility (Figures 2A and 2B). *cop1-4*, *cop1-6*, and *det1-1* seedlings showed enhanced recovery 10 days post-inoculation (Figure 2C), while *COP1-OE* showed sustained susceptibility and poor recovery, which was comparable to those of the defense-compromised *eds1-2* mutant (Feys et al., 2001). Both COP1 and DET1 are critically important for sustaining hypocotyl elongation and its modulation by photoperiod (Gangappa and Kumar, 2017). Consistent with the antagonistic relation between growth and immunity, we found a very strong correlation (*R*^2^ = 0.96) between hypocotyl length and susceptibility to *Pto* DC3000 as a result of altered DET1/COP1 function (Figures 2D and S2). In addition, the COP1/DET1 signaling module suppressed immunity in 4-week-old adult plants (Figure 2E). In support of our above results, the effect of DET1 and COP1 in modulating resistance was more apparent in shorter photoperiods, and the mutants showed no significant difference in resistance compared to the wild-type at constant light (LL) (Figures 2F and 2G). We found a strong genotype × environment (G × E) interaction, as shown by two-way ANOVA, supporting the role of DET1 and COP1 in mediating photoperiod-dependent modulation of immunity (Figures 2F and 2G). Gene expression analyses further supported the role of DET1/COP1 in modulating immunity (Figures 2H–2J). Expression of *PR1*, *PBS3*, and *PAD4* was significantly upregulated in *cop1-4, cop1-6*, and *det1-1* in SD, suggesting that DET1/COP1 signaling negatively regulates defense gene expression (Figures 2H–2J). These results show that the DET1/COP1 signaling module acts to negatively regulate defense gene expression, particularly in SD, albeit with some allele-specific and gene-specific exceptions (Figures 2H–2J). It is likely that while these play an important role in modulating defense gene expression in response to changing photoperiod, additional factors may be required to fully explain the regulatory framework. Nevertheless, the results above clearly show the novel role of DET1/COP1 signaling in immunity.

### PIF4 Plays a Key Role in Day Length-Dependent Modulation of Immunity

**Figure 3:**
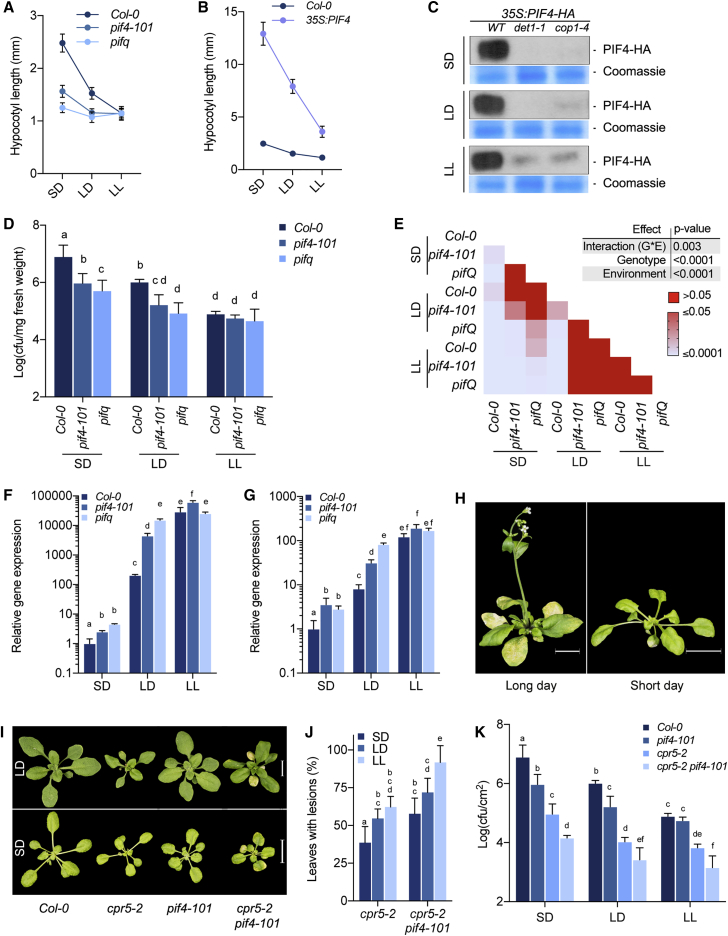
PIF4 Mediates Photoperiod-Dependent Modulation of Immunity (A) Hypocotyl length phenotype of 7-day-old Col-0, *pif4-101*, and *pifq* seedlings grown under SD, LD, and LL conditions (n ≥ 20). (B) Hypocotyl length phenotype of 7-day-old Col-0 and *35S:PIF4-HA* seedlings grown under SD, LD, and LL conditions (n ≥ 20). (C) Immunoblot analysis of PIF4-HA abundance in wild-type, *det1-1*, and *cop1-4* backgrounds under SD, LD, and LL conditions. Seven-day-old seedlings grown at respective conditions were sampled at dawn. Coomassie blue stain gel serves as loading control. (D) Disease resistance phenotype of 12-day-old Col-0, *pif4-101*, and *pifq* plants grown under SD, LD, and LL conditions (n = 6). (E) Two-way ANOVA followed by Tukey’s HSD test of data from (E). (F and G) Gene expression of *PR1* (F) and *PR5* (G) in Col-0, *pif4-101*, and *pifq* seedlings grown on soil under SD, LD, and LL conditions for 12 days (n = 3). Data on Col-0 are the same as those presented in Figures 1D and 1E. Data shown are the means ± SDs of three biological replicates. (H) Representative images showing the photoperiod-dependent lesion-mimic phenotype of *cpr5-2* mutant. Four-week-old plants grown under SD and LD conditions at 22°C are shown. Scale bar, 1 cm. (I) Representative images of 3-week-old Col-0, *cpr5-2*, and *cpr5-2 pif4-101* plants grown under SD and LD conditions, showing the role of PIF4 in photoperiod-dependent modulation of *cpr5-2* phenotypes. Scalebar, 1 cm. (J) Quantification data showing lesion-severity phenotype of 17-day-old 22°C grown *cpr5-2* and *cpr5-2 pif4-101* mutants under different photoperiods, as indicated. At least 14 plants (n ≥ 14) were used in each genotype under respective growth conditions for scoring the phenotype. (K) *Pto* DC3000 disease-resistance phenotype of 12-day-old Col-0, *pif4-101, cpr5-2*, and *cpr5-2 pif4-101* plants grown under SD, LD, and LL conditions (n = 6). The data presented are representative of three independent experiments. Data points with the same letters are statistically not significant based on two-way ANOVA followed by Tukey’s multiple comparison test (p < 0.05).

DET1/COP1 signaling controls photoperiod-dependent growth through PIF4 (Gangappa and Kumar, 2017).PIF4 has also recently been shown to have a role in coordinating growth and immunity (Gangappa et al., 2017). Therefore, we asked whether PIF4 is involved in the coordination of growth and defense in response to photoperiod. Highlighting the role of PIF4 in photoperiod-dependent control of growth, hypocotyl elongation is strongly photoperiod dependent, and loss of PIF function abolished the same both under normal growth conditions and when grown at high-density-mimicking shade (Figures 3A and S3A). Moreover, only a modest difference was observed between *pif4-101* and *pifq* quadruple mutant, which is deficient in the major *PIFs* (*PIF1*, *PIF3*, *PIF4*, and *PIF5*) (Leivar et al., 2008), suggesting that PIF4 is the major player that accelerates growth in response to a shorter photoperiod (Figures 3A and S3A). Accordingly, the very long hypocotyl phenotype of a transgenic line overexpressing PIF4 (35S:*PIF4-HA*) was strongly suppressed by increasing photoperiod (Figure 3B). Further confirming the role of the DET1/COP1 signaling module in controlling PIF4-mediated responses, PIF4 accumulation was severely depleted in both *det1* and *cop1* mutant backgrounds at all photoperiods (Figure 3C). These results suggest that DET1 and COP1 modulate immunity in response to photoperiod, likely through regulating the PIF4 function. Therefore, to test whether PIF4 has any role in day length-mediated modulation immunity, we studied resistance to *Pto* DC3000 in *pif4-101* and *pifq*. Both mutants were significantly more resistant in SD and were moderately more resistant in LD compared to wild-type (Figures 3D and 3E). However, their resistance was comparable to Col-0 in constant light (LL) (Figures 3D and 3E). This is consistent with PIF4 function being photoperiod dependent, in which it is most favored under shorter days and increasing day length inhibiting PIF4 function. Two-way ANOVA showed a strong G × E interaction in determining photoperiod-dependent effect, suggesting a key role for PIF4 in the modulation of plant defense in response to day length (Figures 3D and 3E). More specifically, our data show that increased PIF4 function appears to underlie enhanced susceptibility in SD.

Consistent with its role as a negative regulator of defense (Gangappa et al., 2017), we found that PIF4 modulates the expression of defense marker genes *PR1* and *PR5* in different photoperiods. In *pif4-101* and *pifQ,* these genes were significantly upregulated in SD and LD (Figures 3F and 3G). At LL, loss of PIF function has little or no effect, suggesting the key role that PIFs play in mediating the photoperiod effect. Notably, at SD, *pif4-101* and *pifq* showed comparable gene expression, suggesting that *PIF4* may be playing a predominant role under this condition. Other PIFs could also be contributing to the suppression of these defense genes specifically in longer days (LD and LL), as their expression in *pifq* is significantly increased over *pif4* single mutant (Figures 3F and 3G). The results above show that PIFs play a key role in the modulation of immunity, particularly in enhancing susceptibility under shorter photoperiods.

### PIF4 Mediates the Conditioning of Lesion-Mimic Mutant Phenotypes by Photoperiod

One of the notable examples of light influence on immunity is the modulation of constitutive defense mutant phenotypes (Chaouch et al., 2010, Dietrich et al., 1994). For example, the lesion-mimic phenotype of the mutant *cpr5-2* (*constitutive expresser of pathogenesis-related genes5)* is conditioned by day length (Boch et al., 1998, Bowling et al., 1997). The *cpr5-2* mutant is characterized by spontaneous chlorotic or necrotic lesions, elevated expression of defense genes, and increased resistance to *P. syringae* (Boch et al., 1998, Bowling et al., 1997). Consistent with the previous reports, we found that the lesion phenotype of *cpr5-2* is strongly influenced by day length. The necrotic lesions were more severe in LD than under SD conditions (Figures 3H and S3B). In line with the above results, the modulation of *cpr5-2* phenotypes by day length was PIF4 dependent. First, we found that the lesion-mimic phenotype of *cpr5-2* was enhanced in *cpr5-2 pif4-101* double mutant, even in SD (Figures 3I, 3J, and S3C). Second, resistance of *cpr5-2* mutant to *Pto* DC3000 was also modulated by photoperiod, as the *cpr5-2* plants grown in LD and LL were significantly more resistant than the plants grown at SD and LD, respectively (Figure 3K). Consistent with the role of PIF4 in modulating immunity, *pif4-101* enhanced the resistance phenotype of *cpr5-2* (Figure 3I). *cpr5-2 pif4-101* retained enhanced resistance at a shorter photoperiod, a condition in which the *cpr5-2* single mutant was strongly suppressed (Figure 3K). Collectively, the data presented here confirm a role for PIF4 in day length-dependent modulation of immunity.

### The DET1/COP1 Signaling Module Regulates Temperature Sensitivity of Defense Responses

**Figure 4:**
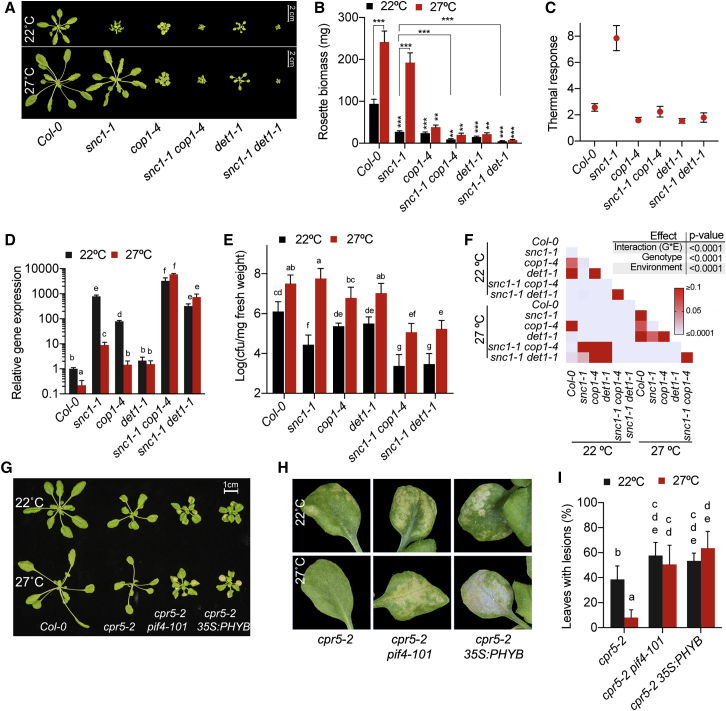
DET1 and COP1 Regulate Temperature Sensitivity of Defense Responses (A) Elevated temperature-mediated suppression of *snc1-1* phenotypes are COP1 and DET1 dependent. Representative images of 4-week-old Col-0 single and double mutants grown at 22°C and 27°C SD. Scale bar, 2 cm. See also Figure S4A for original images. (B) Rosette biomass (n ≥ 8) of plants shown in (A). (C) Thermal response (27°C versus 22°C) of the plant rosette biomass for the data shown in (B). (D) Expression of defense marker genes *PR1* in 18-day-old Col-0, *snc1-1, cop1-4, snc1-1 cop1-4, det1-1*, and *snc1-1 det1-1* at 22°C and 27°C SD (n = 3). Data shown are the means ± SDs of three biological replicates. (E) Resistance to *Pto* DC3000 of 3-week-old Col-0, *snc1-1, cop1-4, snc1-1 cop1-4, det1-1*, and *snc1-1 det1-1* at 22°C and 27°C SD (n = 8), showing the requirement of DET1 and COP1 in thermosensory suppression of immunity in *snc1-1*. (F) Two-way ANOVA with Tukey’s multiple comparison test of data from (E). (G) Elevated temperature-induced suppression of *cpr5-2* lesion-mimic phenotype is PIF4 dependent. Representative rosette picture of 3-week old Col-0, *cpr5-2*, *cpr5-2 pif4-101*, and *cpr5-2 35S:PHYB* genotypes grown under SD conditions at 22°C and 27°C. (H) Enlarged images of individual leaves from plants shown in (G), showing severity of necrotic/chlorotic lesions. Wild-type Col-0 is not shown because it did not show any lesion phenotypes under either condition. (I) Lesion-mimic phenotype of 17-day-old *cpr5-2* and *cpr5-2 pif4-101* and *cpr5-2 35S:PHYB* genotypes grown under SD conditions at 22°C and 27°C. At least 14 plants (n ≥ 14) were used in each genotype under respective growth conditions for scoring the phenotype. The data (means ± SDs) presented are the representative of three independent biological experiments. The data points with the same letters are statistically not significant based on two-way ANOVA followed by Tukey’s multiple comparison test (p < 0.05). Asterisks indicate statistically significant (Student’s t test; ^∗∗^p ≤ 0.01, ^∗∗∗^p ≤ 0.001) differences from Col-0 in corresponding temperature conditions or between indicated pairs of genotypes.

The data presented above show that the COP1/DET1-PIF4 signaling module plays a key role in the modulation of growth and immunity by photoperiod. We have recently shown that the DET1/COP1-PIF4 module controls thermosensory elongation growth in response to photoperiod. While promoting growth, elevated ambient temperatures suppress plant immunity, in which PIF4 has been shown to play a major role (Gangappa et al., 2017, Paik et al., 2017). Therefore, we examined the possible role of DET1/COP1 signaling in the modulation of defense by temperature. Supporting the above results, we found that COP1 and DET1 modulate defense mediated by the nucleotide-binding and leucine-rich repeat (NB-LRR) proteins such as SNC1 (Suppressor of npr1-1 Constitutive 1). The gain-of-function mutant *snc1-1* has constitutive defense activation, severely reduced growth, increased expression of defense genes, and enhanced resistance to *Pto* DC3000 (Zhang et al., 2003). Both *det1*-*1* and *cop1*-*4* mutations strongly enhanced *snc1-1* phenotypes (Figures 4A, 4B, and S4A). Accordingly, the expression of *PR1* was significantly enhanced in *snc1-1 cop1-4*, but not in *snc1-1 det1-1* (Figure 4D), when compared to *snc1-1* at 22°C. Moreover, resistance to *Pto* DC3000 was significantly enhanced in both of the double mutants compared to *snc1-1* at 22°C (Figures 4E and 4F).

Enhanced immunity and the consequent growth defects associated with the *snc1-1* mutation are suppressed by moderately elevated temperatures such as 27°C (Gangappa et al., 2017, Zhu et al., 2010). To test whether DET1 and COP1 are also involved in temperature sensitivity of defense, we studied the growth and immunity phenotypes at elevated temperature. We found that both *det1-1 snc1-1* and *cop1-4 snc1-1* double mutants showed little or no suppression of growth defects at 27°C (Figures 4A–4C and S4A–S4C). Moreover, *PR1* expression in both *det1-1 snc1-1* and *cop1-4 snc1-1* mutants is either maintained or significantly enhanced even at 27°C in comparison to 22°C (Figure 4D). Its expression is significantly increased in comparison with *snc1-1* at 27°C. Accordingly, *det1-1 snc1-1* and *cop1-4 snc1-1* double mutants showed significantly enhanced resistance to *Pto* DC3000 at 27°C, while the single mutants were susceptible to the level similar to wild-type (Figures 4E and 4F).

Complementary to these results, we also found that the lesion-mimic phenotype of the *cpr5-2* mutant was suppressed at 27°C, which was PIF4 dependent. The *cpr5-2 pif4-101* double mutant showed a strong lesion phenotype even at 27°C (Figures 4G–4I). The photoreceptor phytochrome B (PhyB) negatively regulates PIF4 and other PIFs at the protein level through promoting light-dependent protein degradation and by inhibiting their function. Accordingly, overexpression of *PHYB* in the *cpr5-2* background also led to a strong enhancement of the lesion-mimic phenotype at 22°C, which was maintained even at 27°C, mimicking *cpr5-2 pif4-101* (Figures 4G–4I), while neither *pif4-101* nor *35S:PHYB* (Ádám et al., 2013) showed any lesion-mimic phenotypes (Figure S4D). These results show that along with PIF4, DET1/COP1 signaling plays an essential role in the suppression of immunity at elevated temperatures. Our data confirm that the DET1/COP1-PIF4 module plays an important role in mediating the effects of seasonal signals such as photoperiod and temperature for the modulation of growth and defense.

Our findings extend the understanding of the mechanistic framework of environmental signal integration. This could act as a coordinated signal integration hub underlying seasonal phenological responses. DET1/COP1 signaling promotes PIF-mediated growth primarily during the dark, while suppressing defense. On the contrary, phytochrome-mediated repression of PIF function in light inhibits growth while promoting immunity (Paik et al., 2017). Photoreceptor signaling has been previously shown to be important in plant defense responses (Ballaré, 2014, Griebel and Zeier, 2008, Jeong et al., 2010, Wu and Yang, 2010). It has been shown that the circadian clock controls the timing of plant defense responses (Wang et al., 2011); therefore, it is likely that the photoperiod-mediated modulation of immunity we observe is at least in part due to its effect on the circadian clock. DET1/COP1 has been implicated in the control of the circadian clock (Lau et al., 2011). It remains to be seen to what extent the photoperiod modulation of growth and immunity by the DET1/COP1-PIF4 module is through their potential influence on the circadian clock. Our findings on the involvement of DET1/COP1 in plant immunity are also supported by the recent implication of the SUMO E3 ligase SIZ1 in temperature modulation of immunity (Hammoudi et al., 2018). SIZ1 has been shown to promote the function of COP1 through enhancing its ubiquitin ligase activity (Lin et al., 2016), providing a mechanistic link.

It has been previously shown that environmental modulation of plant immunity is underlain by the trade-off between growth and immunity (Alcázar and Parker, 2011, Gangappa et al., 2017, Zhu et al., 2010). The coordination of growth and immunity in response to seasonal signals by this common signaling module provides a robust mechanism for the control of important traits in nature for optimizing fitness. Phenological and physiological responses, including immunity, are also driven by the circadian clock (Wang et al., 2011) and the consequent changes in metabolism (Dodd et al., 2005). The contribution of these factors to the seasonal modulation of growth and defense would also be important factors to consider. Future studies will determine whether this emerging signal integration module plays an important role in coordinating these processes. Elucidating the fundamental principles that define seasonal responses of plants in a true ecological context will be key in protecting biodiversity and ensuring food security.

## STAR★Methods

### Key Resources Table

**Table:** 

REAGENT or RESOURCE	SOURCE	IDENTIFIER
**Bacterial and Virus Strains**		

*Pseudomonas syringae* pv. Tomato (*Pto*) DC3000	Lab Stock	N/A
Pto DC3000-lux	Fan et al., 2008	N/A

**Experimental Models: Organisms/Strains**		

*Arabidopsis thaliana* accession*: Col-0*	Lab stock	N/A
*pif4-101*	Lab stock	Garlic_114_G06
*cop1-4*	Holm et al., 2001	N/A
*cop1-6*	Holm et al., 2001	N/A
*det1-1*	NASC	N6158
*snc1-1*	Zhang et al., 2003	N/A
*cpr5-2*	NASC	N3770
*cpr5-2 pif4-101*	This paper	N/A
*cpr5-2 35S:PHYB*	This paper	N/A
*snc1-1 cop1-4*	This paper	N/A
*snc1-1 det1-1*	This paper	N/A
*eds1-2*	Feys et al., 2001	N/A
*PR1::LUC*	*NASC*	N9850
*35S:PIF4-HA*	Nozue et al., 2007	N/A
*35S:PHYB*	Ádám et al., 2013	N/A
*35S:COP1 (COP1-OE)*	Holm et al., 2001	N/A

**Chemicals, Peptides, and Recombinant Proteins**		

Murashige and skoog’s medium	Sigma	M5519
Sucrose	Sigma	S7903
MES	Sigma	M8250
Agar	Sigma	A1296
Yeast extract	BD Biosciences	212750
Peptone	BD Biosciences	211677
Bacto Agar	BD Biosciences	214010
Glycerol	Fisher Scientific	10795711
RNase-Free DNase Set	QIAGEN	79254
Oligo(dT)12-18 Primer	Invitrogen	18418-012
dNTPs	Invitrogen	10297-018
RNasin® Ribonuclease Inhibitors	Promega Carporation	N2111
Anti-HA HRP antibody	Miltenyi Biotech	130-091-972; RRID:AB_871936

**Critical Commercial Assays**		

RNeasy Plant Mini Kit	QIAGEN	74904
Superscript III reverse transcriptase assay	Invitrogen	18080044
Light Cycler 480 SYBR Green I Master	Roche	04-887-352-001

**Oligonucleotides**		

Primers for genotyping, see Table S1	This study	N/A
Primers for qPCR, see Table S1	This study	N/A

**Software and Algorithms**		

Graphpad PRISM7.0	GraphPad Software Inc.	N/A
ImageJ	NIH	https://imagej.nih.gov/ij/

### Contact for Reagent and Resource Sharing

Further information and requests for resources and reagents should be directed to and will be fulfilled by the Lead Contact, Vinod Kumar (vinod.kumar@jic.ac.uk).

### Experimental Model and Subject Details

#### Arabidopsis thaliana

The model used in this study is *Arabidopsis*. The wild-type used is the accession Columbia (Col-0). The various mutants used in this study have been described in the Key Resources Table.

### Method Details

#### Plant materials and growth conditions

All the seed material used in this study are in the Columbia background. The mutants and transgenic lines used in the study are listed in the Key Resources Table. Surface-sterilized (70% ethanol + 0.5% Triton X-100) seeds were plated onto ½ MS medium, and stratified for 3 days at 4°C in dark and transferred to 22°C short-day photoperiod (SD; 8h light/16 h dark) for germination. Later they were shifted to respective light (short-day, long-day, constant light) or temperature (22°C or 27°C) regimes as indicated. Unless otherwise mentioned plants grown at a Photosynthetic Photon Flux Density (PPFD) of 150 μmol m^-2^ s^-1^ were used for the experiments. For testing the possible influence of photosynthetically active radiation in modulating immunity, LD conditions were supplied with either 150 μmol m^-2^ s^-1^ (LD^150^) or 75 μmol m^-2^ s^-1^ (LD^75^) were used, as specified in the text.

#### Generation of double mutants

The double mutants *cpr5-2 pif4-101* and *cpr5-2 35S:PHYB* were generated by crossing *cpr5-2* to *pif4-101* and *35S:PHYB*, respectively. The F_2_ seeds were screened at 27°C to identify short hypocotyl seedlings corresponding to *pif4-101* and *35S:PHYB*. In adult stage, homozygous double mutants were identified through genotyping for *pif4*-101 (primers 85, 213 and 214), phenotyping (necrotic lesions phenotype) for *cpr5-2*. Seeds of approximately 20 F_2_ plants with *cpr5-2* (lesion-mimic) phenotype were screened on MS Kanamycin to identify homozygous plants for *35S:PHYB* transgene. For the generation of *snc1-1 cop1-4* and *snc1-1 det1-1* double mutants, *snc1-1* was crossed to *cop1-4* and *det1-1*, respectively. F_2_ seeds were screened at 27°C and seedlings with short hypocotyls corresponding to *cop1-4* and *det1-1* were identified and transferred to soil. In adult stage plants with *snc1-1* were identified and further confirmed for homozygosity for *snc1-1* mutation through genotyping (primers 124 and 125).

#### Pathogen assays

For bacterial resistance assays, unless otherwise mentioned, plants were grown in controlled growth cabinets at 22°C with a PPFD of 150 μmol m^-2^ s^-1^ and relative humidity of 70%. Three-week-old plants or as specified in the text were spray inoculated using *Pseudomonas syringae pv. tomato* DC3000 (OD_600_ = 0.002) in 10 mM Mgcl2 and 0.04% SilwetL-77. Here adult plants were analyzed, three-days-post inoculation, leaf discs from three leaves were collected from at least 6 plants. Bacteria were recovered from leaf discs or whole seedlings as specified in respective legends in 10mM MgCl_2_ with 0.01% Silwett L-77 at 28°C. In case of seedlings or experiments involving genotypes with small plants size such as det1 snc1-1 and cop1 snc1-1 bacteria were collected from aerial parts of the plants and fresh weights were recorded. Serially diluted culture was spotted on to plates containing King’s medium with Rifampicin (50 μg/ml) and Kanamycin (50μg/ml), and incubated for two days at 28°C. Bacterial cfu were normalized to unit area or to fresh weight as specified in the text or in Figure legends.

For luminescence-based estimation of bacterial infection, seedlings were grown for seven days in SD at 22°C, and then spray inoculated with the luminescent *Pto* DC3000-lux. Three-days-post spray inoculation, seedlings were aligned on MS plates and scanned for luminescence using a photon counting camera (Photek). Total photon emission from individual seedlings were extracted and were normalized to unit area.

#### Luciferase imaging assay

Luciferase imaging assay has been carried out for measuring *In planta* promoter-reporter activity of *PR1* promoter. For analyzing this, *PR1:LUC* transgenic line seedlings were grown in SD for five days and transferred to LD and constant light conditions for additional two days. On seventh day seedlings were treated with 1 mM beetle luciferin and immediately scanned for luminescence for specific time period. Total luminescence was then normalized to fresh weight of the seedlings in corresponding conditions, and the *PR1:LUC* activity was expressed as Photon count/mg fresh weight. For analyzing the effect of photosynthetically active radiation on *PR1:LUC* expression, seedlings were grown in SD with 150μmol m^-2^ s^-1^ (SD^150^) for 3 days and then transferred to LD conditions with 150 μmol m^-2^ s^-1^ (LD^150^) or 75 μmol m^-2^ s^-1^ (LD^75^) of light intensity.

#### RNA extraction and gene expression analysis by qPCR

For gene expression analysis using quantitative-PCR (qPCR), RNA was extracted using RNeasy Plant mini kit (QIAGEN) with on-column DNase I digestion according to the manufacturer’s instructions. RNA was quantified using NanoDrop, and approximately 2.0 μg of total RNA was converted into cDNA using Superscript III reverse transcriptase (Invitrogen) and oligo dT according to the manufacturer instructions. 2 μL of 1:20 diluted cDNA was used for qPCR using 2X SYBR Green Master Mix kit in Roche Lightcycler 480. qPCR experiments were performed in Light Cycler LC480 using Light Cycler 480 SYBR Green I Master (Roche). *EF1∝* (*AT5G60390*) was used as internal control for normalization. Details of the oligo nucleotide primers used are provided below.

#### Quantification of lesion phenotype

For the quantification of lesion phenotype, *cpr5-2*, *cpr5-2 pif4-101* and *cpr5-2 35S:PHYB* genotypes were grown in respective photoperiod and temperature conditions for 17-days with Photosynthetic Photon Flux Density of 150 μmol m^-2^ s^-1^. In each plant, total number of number of leaves and leaves with visible chlorotic and/or necrotic lesions were scored. At least 14 (n ≥ 14) plants were used in each genotype under respective growth condition. Percentage of leaves with lesions were then calculated and plotted.

#### Hypocotyl measurement

For measuring hypocotyls, surface sterilized seeds were stratified at 4°C for 3 days and germinated at 22°C SD on 1∕2 MS media for 7 days before they were aligned on 1% agar plate and imaged using stereomicroscope. Hypocotyl length (using at least 20 seedlings) was measured using NIH ImageJ software (https://imagej.nih.gov/ij).

#### Sampling of biological material

All experiments were performed with at least 3 biological replicates as indicated at respective sections. For analyzing gene expression in soil-grown seedlings, aerial parts of several seedlings (∼20) were pooled and were considered as one biological replicate. For plate-grown seedlings, entire seedlings were collected. For pathogen resistance assays, discs from three leaves were collected from at least 6 plants for collecting bacteria prior to cfu estimation. In case of pathogen assays using young seedlings, green aerial parts (cotyledons and leaf) were collected from ∼10 seedlings were collected and fresh weight was recorded before bacteria were extracted for cfu estimation. All experiments were repeated at least thrice and data from a representative experiment is presented.

### Quantification and Statistical Analysis

All the experiments were repeated at least twice, and data from a representative experiment are shown. The experiments related to gene expression analysis were done in three biological replicates. The data were subjected to statistical analysis using either Student’s t test (two-tailed) or Two-way ANOVA analysis with Tukey’s multiple comparison test as specified in individual figure legends. In all graphs, error bars are standard deviation.

## Supplemental Information

Document S1. Figures S1–S4 and Table S1

Document S2. Article plus Supplemental Information
