# Supplementary material for: DET1 and COP1 Modulate the Coordination of Growth and Immunity in Response to Key Seasonal Signals in Arabidopsis
Source: Cell Rep. 2018 Oct 2;25(1):29–37.e3. doi: 10.1016/j.celrep.2018.08.096 (PMC6180345; doi:10.1016/j.celrep.2018.08.096)
Supplement: Document S1. Figures S1–S4 and Table S1 [file mmc1.pdf]

Cell Reports, Volume *25*

Supplemental Information

DET1 and COP1 Modulate the Coordination of Growth and Immunity in Response to Key Seasonal Signals in *Arabidopsis*

Sreeramaiah N. Gangappa and S. Vinod Kumar

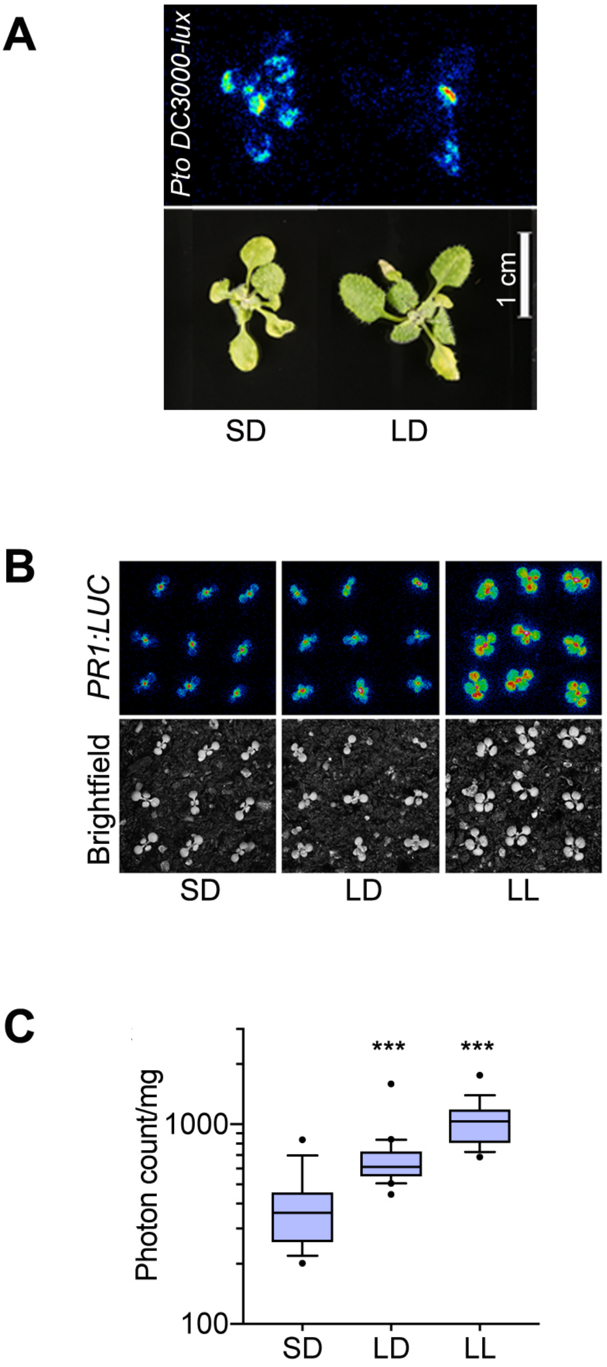

Figure S1. Modulation of immunity by Photoperiod. Related to Figure 1

(A) Three-weeks old short-day (SD) and long-day (LD) grown plants were spray inoculated with luminescent *P. syringae pv*. tomato (*Pto)* DC3000. Note that SD grown plants harbor more bacteria as seen in increased luminescence than LD grown plants.

(B) Images of *PR1::LUC* seedlings grown under SD for four days before being shifted to LD and LL for additional two days and imaged for luminescence.

(C) Quantified luminescence intensity normalized to fresh wt. of the seedlings (n = 8) for *PR1::LUC* expression shown in (A).

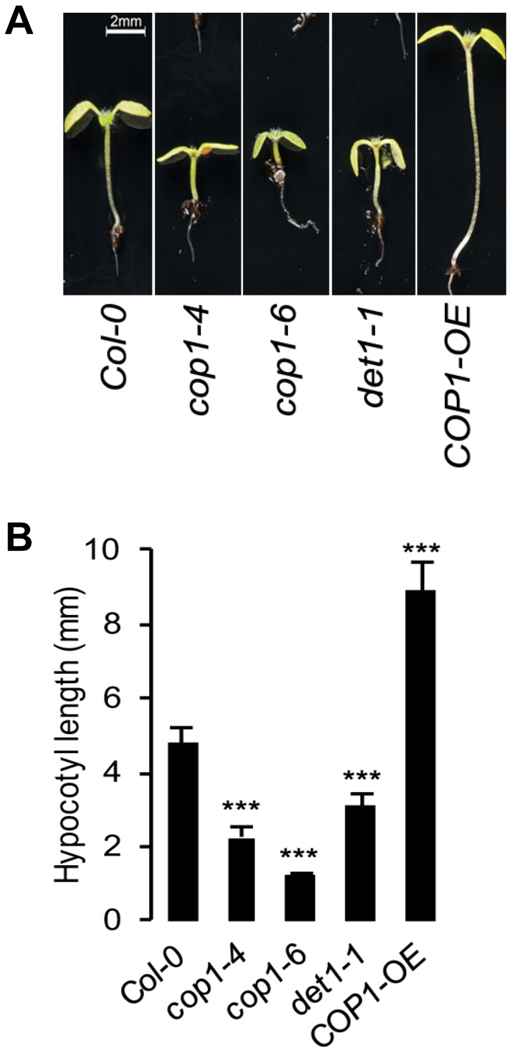

Figure S2. Hypocotyl elongation phenotypes of *cop1* and *det1* mutants. Related to Figure 2.

(A, B) Representative seedling picture (A) and hypocotyl elongation phenotype (B) of Col-0, *cop1-4*, *cop1-6*, *det1-1* and *COP-OE.* Seedlings grown in SD for 10 days before imaging and quantification.

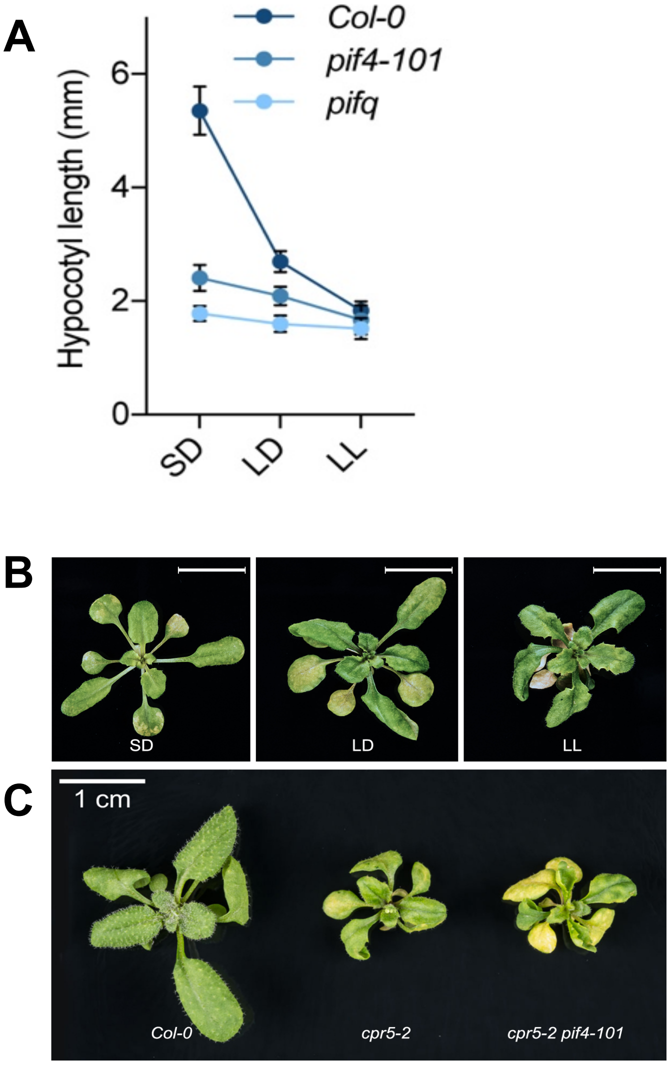

Figure S3. Role of *PIF4* in photoperiod-dependent modulation of growth and immunity. Related to Figure 3.

(A) Hypocotyl elongation data (mean ± SD; n ≥ 20) of 7-day-old Col-0, *pif4-101* and *pifq* seedlings grown under SD, LD and LL with high density planting mimicking shade.

(B) Representative images of four-week old *cpr5-2* mutant showing lesion mimic phenotype from 22°C grown SD, LD, and LL grown plants. Plants were grown in SD for three weeks before shifting to LD and LL for a week.

(C) Representative pictures of three-week old plants grown in constant light at 22°C showing enhancement of *cpr5-2* lesion mimic phenotype by *pif4-101*.

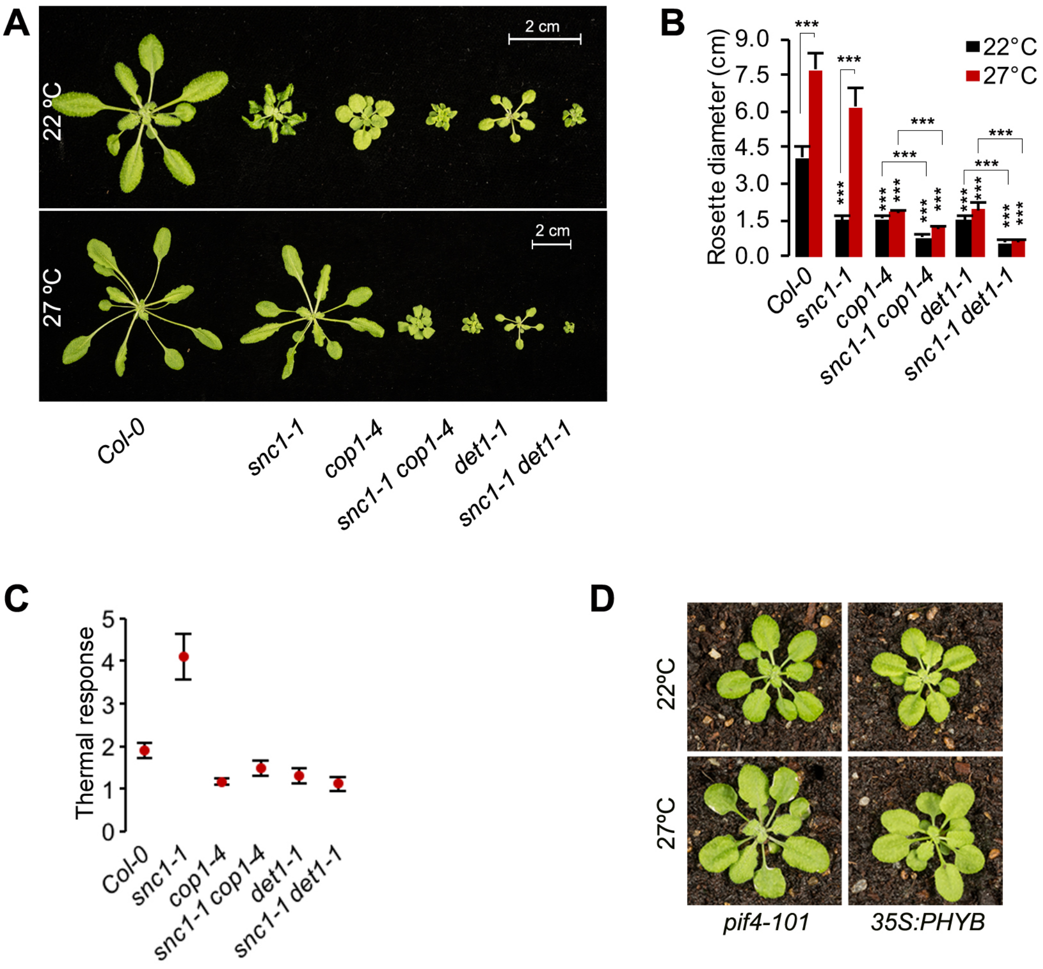

Figure S4. COP1/DET1 are essential for the temperature mediated suppression of defense responses. Related to Figure 4.

(A) Original images corresponding to Fig. 4A, depicting the role of DET1 and COP1 in the modulation of immunity-related phenotypes of *snc1-1* by temperature. To better represent the effect of temperature as well as the *det1-1* and *cop1-4* mutations on *snc1-1* phenotype, plant images from both 22ºC and 27ºC are shown in same scale in Fig. 4A (B, C) COP1 and DET1 are essential for the temperature mediated suppression of *snc1-1* growth phenotype. Data showing the rosette diameter (B) and the temperature responsiveness (C) of the same for Col-0, *snc1-1*, *cop1-4*, *det1-1*, *snc1-1 cop1-4* and *snc1-1 det1-1* mutants grown in SD at 22°C and 27°C for four-weeks.
(D) Rosette pictures of three-week-old *pif4-101* and *35S:PHYB* plants grown at 22°C and 27°C under short days shows no lesion phenotypes.

Data presented are mean ± SD; $^{*}p \leq 0.05$, $^{**}p \leq 0.01$, $^{***}p \leq 0.001$ (Student's t-test) significantly different from Col-0 or between indicated pairs of genotypes.

Table S1. List of oligonucleotides used in this study. Related to Figures 1-4.

Gene Name	Oligo No.	Oligo Sequence (5'…………..3')	Purpose
PIF4-LP	213	*AATACATTTTGCAGGCAATCG*	Genotyping *pif4-101*
PIF4-RP	214	*CGTAATGAAGTTGCACGTTTACTC*	Genotyping *pif4-101*
PIF4-LB	85	*TTCATAACCAATCTCGATACAC*	T-DNA specific oligo for genotyping *pif4-101*
SNC1-FP	124	*ATACGTTTGCCATTCGAGGA*	Genotyping *snc1-1*
SNC1-RP	125	*ACCAGAGTTCCTTCCCACAG*	Genotyping *snc1-1*
PR1-F	18	*ACCAGGCACGAGGAGCGGTA*	Q-PCR
PR1-R	19	*TCCCCGTAAGGCCCACCAGA*	Q-PCR
PR5-F	20	*ACCCACAGCACAGAGACACACA*	Q-PCR
PR5-R	21	*TGGCCATAACAGCAATGCCGC*	Q-PCR
PAD4-F	315	*TGGTGACGAAGAAGGAGGTT*	Q-PCR
PAD4-R	316	*TCCATTGCGTCACTCTCATC*	Q-PCR
PBS3-F	323	*TGAGTCAAGCGAAGCTCGTA*	Q-PCR
PBS3-R	324	*ATCGATCCGTCTTTGAATCG*	Q-PCR
